# Integrating simulated and experimental data to identify mitochondrial bioenergetic defects in Parkinson’s Disease models

**DOI:** 10.1371/journal.pone.0339326

**Published:** 2026-01-05

**Authors:** Sandeep Chenna, Alvin Joselin, Pierre Theurey, Daniele Bano, Paola Pizzo, Maria Ankarcrona, David S. Park, Jochen H. Prehn, Niamh M. C. Connolly

**Affiliations:** 1 Centre for Systems Medicine, Department of Physiology and Medical Physics, RCSI University of Medicine and Health Sciences, Dublin, Ireland; 2 Department of Clinical Neurosciences, Hotchkiss Brain Institute, University of Calgary, Calgary, Canada; 3 Department of Biomedical Sciences, and Centro Studi per la Neurodegenerazione (CESNE), University of Padua, Padua, Italy; 4 Neuroscience Institute, National Research Council (CNR), Padua, Italy; 5 German Center for Neurodegenerative Diseases (DZNE), Bonn, Germany; 6 Center for Alzheimer Research, Division of Neurogeriatrics, Department of Neurobiology Care Sciences and Society, Karolinska Institutet, Stockholm, Sweden; 7 FutureNeuro Research Ireland Centre, RCSI University of Medicine and Health Sciences, Dublin, Ireland; University of Tartu, ESTONIA

## Abstract

Mitochondrial bioenergetics are vital for ATP production and are associated with several diseases, including Parkinson’s Disease (PD). Here, we simulated a computational model of mitochondrial ATP production to interrogate mitochondrial bioenergetics under physiological and pathophysiological conditions, and provide a data resource that can be used to interpret mitochondrial bioenergetics experiments. We first characterised the impact of several common electron transport chain (ETC) impairments on experimentally-observable bioenergetic parameters. We then established an analysis pipeline to integrate simulations with experimental data and predict the molecular defects underlying experimental bioenergetic phenotypes. We applied the pipeline to data from PD models. We verified that the impaired bioenergetic profile previously measured in *Parkin* knockout (KO) neurons can be explained by increased mitochondrial uncoupling. We then generated primary cortical neurons from a *Pink1* KO mouse model of PD, and measured reduced oxygen consumption rate (OCR) capacity and increased resistance to Complex III inhibition. Here, our pipeline predicted that multiple impairments are required to explain this bioenergetic phenotype. Finally, we provide all simulated data as a user-friendly resource that can be used to interpret mitochondrial bioenergetics experiments, predict underlying molecular defects, and inform experimental design.

## Introduction

Mitochondrial ATP production (oxidative phosphorylation) is achieved through the oxygen-dependent maintenance of a proton circuit by the mitochondrial electron transport chain (ETC), the F_1_F_o_ ATP synthase, and proton leaks [1]. The mitochondrial ETC is a series of four multi-subunit complexes (complexes I-IV) embedded in the inner mitochondrial membrane. These complexes, via the pumping of protons (H^+^) out of the mitochondrial matrix coupled with electron transport through the complexes, serve to generate and maintain the electrochemical proton-motive force (Δ*p*), composed of the H^+^ concentration gradient (ΔpH_m_) and the mitochondrial membrane potential (ΔΨ_m_). This gradient drives ATP synthesis by enabling H^+^ flow back into the matrix through the F_1_F_o_ ATP synthase, and also maintains metabolite transport and ion homeostasis. H^+^ can also flow into the matrix through H^+^ leaks, bypassing the F_1_F_o_ ATP synthase and contributing to mitochondrial uncoupling, the dissociation between electron transport and its use to drive ATP synthesis [2,3]. Substrates generated by the tricarboxylic acid (TCA) cycle, such as NADH and FADH, serve as electron donors to the ETC.

Altered mitochondrial bioenergetics have been associated with a wide variety of pathologies, including in most neurodegenerative diseases (NDs), and often emerge prior to clinical symptoms [4–7]. In this regard, neurons are particularly susceptible to disruptions in mitochondrial ATP production, as synaptic activity relies heavily on oxidative phosphorylation for ATP supply [8]. For example, deficiency in the expression and activity of Complex I is commonly reported in Parkinson’s Disease (PD) [9–12], following the original observation of Parkinsonian symptoms in a group of people exposed to the Complex I inhibitor 1-methyl-4-phenyl-1,2,3,6-tetrahydropyridine (MPTP) [13,14]. In Alzheimer’s, dysfunctions of the F1Fo ATP synthase and other ETC complexes have been reported [15], as well as reduced glycolysis, substrate supply and TCA cycle capacity [16–18]. Despite expanding knowledge, an understanding of the cause and effect of mitochondrial bioenergetic dysfunction in pathology is lacking.

Mitochondrial bioenergetic function can be evaluated experimentally through measurement of key parameters such as oxygen consumption rate (OCR), Δψ_m_, mitochondrial NAD(P)H or ATP [19,20], and the response of these parameters to specific inhibitors, such as Rotenone or Antimycin A (to inhibit complex I and complex III respectively), provides insights into the function of distinct segments of the system. These parameters are also disrupted in cellular and animal models of NDs e.g., [17,21–23]. Given the intrinsic complexity of mitochondrial bioenergetics, however, experiments require careful interpretation [20] and elucidating the molecular causes underlying bioenergetic dysfunction requires strong knowledge of mitochondrial physiology. Measurement of decreased maximal ETC capacity in respirometry experiments, for instance, could be caused by an underlying defect in any of the ETC complexes, a reduction in proton leak across the membrane (which reduces the impact of pharmacological uncoupler), or a reduced substrate supply to the ETC. Optimal experimental design to pinpoint such dysfunction is vital.

Computational models, based on mechanistic details of relevant pathways, can aid in such data interpretation and experimental design, and have been used to establish causal relationships of bioenergetic defects in NDs [24–27]. Based on these considerations, we developed a computational pipeline to integrate experimental and simulated data and to predict the molecular defects underlying bioenergetic impairments in PD models. We identified reduced bioenergetic function in primary neurons from *Pink1* knockout mice, and applied the integrated pipeline to predict that a combination of defects is required to explain this bioenergetic phenotype. All simulated data are openly provided and available to aid with experimental interpretation and design.

## Methods

### Computational model of the mitochondrial electron transport chain

**Fig 1:**
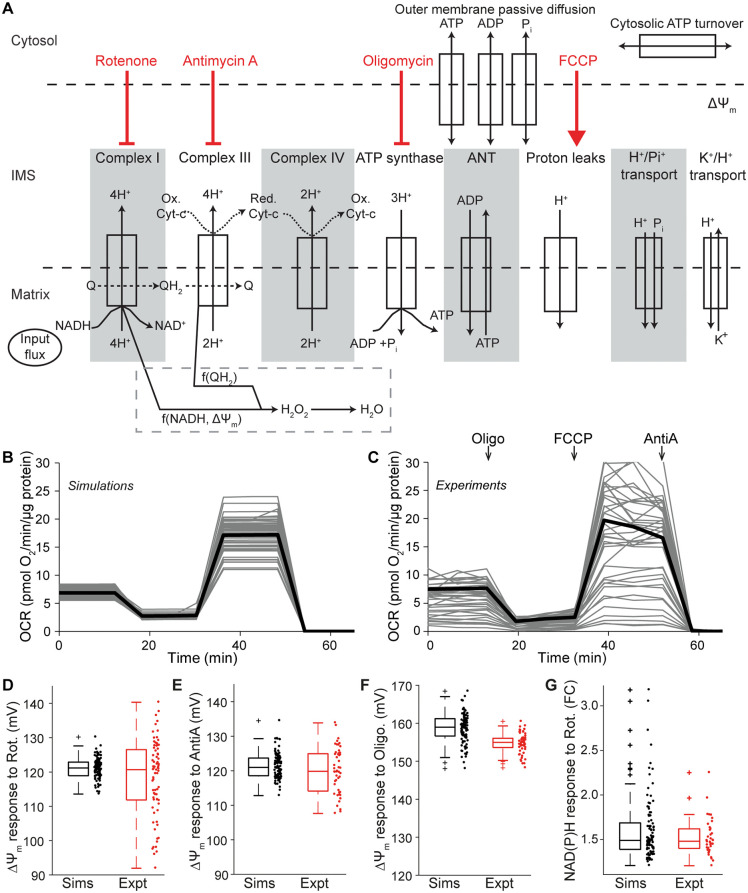
Schematic of the components and fluxes included in the computational model, and comparison of model simulations with experimental measurements. **(A)** Figure adapted from [26]. Addition of pharmacological modulators are simulated by inhibiting (red flat-headed arrows) or increasing (red arrow) the activity of the indicated model component, and arrows point to the text label of the affected model component. We assume the rapid conversion of superoxide to hydrogen peroxide (H_2_O_2_; not shown). ANT: adenosine nucleotide transferase; ΔΨ_m_: mitochondrial membrane potential; H^+^: protons; H_2_O_2_: Hydrogen peroxide; IMM: inner mitochondrial membrane; IMS: inter-membrane space; OMM: outer mitochondrial membrane; P_i_: Phosphate; Q: Ubiquinone; QH_2_: Ubiquinol. **(B,C) (B)** Model simulations of the mitochondrial stress test closely resembled **(C)** experimental measurements of the oxygen consumption rate (OCR) in primary cortical neurons. OCR in **(C)** was measured in wildtype primary cortical neurons at baseline and following addition of Oligomycin (Oligo, 2 μg/ml), FCCP (0.5 μM) and AntimycinA (AntiA, 1 μM), as described in [17], with nonmitochondrial respiration subtracted. Panel C reproduced from [17]. Traces represent individual simulations or wells, and the mean of all traces is shown in black. **(D-G)** The simulated response (Sims) of ΔΨ_m_ to **(D)** Rot, **(E)** AntiA and **(F)** Oligo, as well as the **(G)** simulated response of NAD(P)H to Rot (foldchange, FC) also resembled experimental measurements (Expt; data reproduced from [17]).

The computational model was adapted from a model originally developed by Beard [28] and further extended. The model is a flux-based, thermokinetic, ordinary differential equation model of the mitochondrial electron transport chain (ETC) and ATP production (Fig 1) and is thermodynamically, mass and charge balanced. The model has been used to analyse isolated cardiac and liver mitochondria, *in vivo* skeletal muscle, intact cancer cells, and primary neurons [17,29–31]. The model simulating physiological conditions (PC; in the absence of any impairments) was calibrated to various measurements in non-transgenic primary cortical neurons [17], and was recently expanded to include ETC-derived reactive oxygen species (ROS) metabolism (H_2_O_2_) [26]. We have utilised a relatively simplified model to enable faster calibration, lower computational burden, and easier interpretation by experimentalists. While inclusion of additional detail may capture a broader range of biological processes, we focussed on key relationships and variables to provide accessible and meaningful explanations and predictions.

The model comprises three compartments (mitochondrial matrix, inter-membrane space, cytosol) and the major model components include the ETC complexes CI, CIII, CIV, the F_1_F_o_ ATP synthase (F1), proton leak across the inner mitochondrial membrane (Hle), nucleotide, ion, proton (H^+^) and substrate transport across the mitochondrial membranes, a coarse-grained model of cytosolic ATP consumption/production [31] and ETC-mediated H2O2 metabolism (Fig 1). The model input is provided by a dehydrogenase flux (DH), encompassing NADH/substrate supply upstream of the mitochondrial ETC. CII is considered together with CI, and CII substrates are embedded in this input dehydrogenase flux. Model outputs are represented by 22 state variables, or bioenergetic parameters, including cytosolic and mitochondrial ATP, mitochondrial membrane potential (ΔΨ_m_), mitochondrial NADH (representing redox status [NADH/NAD^+^]) and H_2_O_2_ concentrations. The rate of change of these bioenergetic parameters is defined by the flux through/activity of the modelled components. For example, the simulated NADH concentration at a given timepoint is determined by the combined activities of the NADH production reaction (model input) and the NADH consumption reaction (Complex I). The model was simulated in MATLAB (2017a). Model equations, parameter values, and initial conditions are provided in Supp Methods.

**Fig 2:**
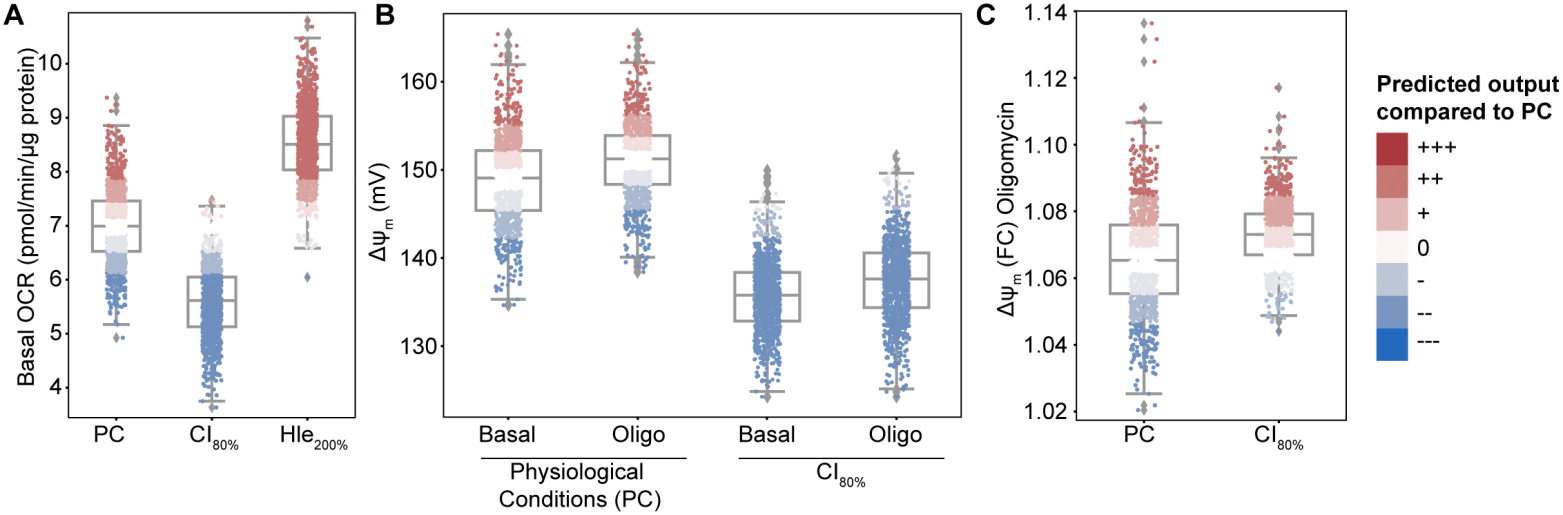
Simulating impairments in model components provides mechanistic insight. **(A)** Basal OCR simulated in physiological conditions (PC; no simulated impairment), in the presence of a complex I impairment (CI_80%_; CI activity = 80% PC activity), and in the presence of increased proton leak (Hle_200%_; Hle activity = 200% PC activity). **(B)** Mitochondrial membrane potential (ΔΨ_m_) simulated at baseline and following the addition of Oligomycin (Oligo), in physiological conditions (left two box-plots) and with a CI impairment (CI_80%_; right two box-plots). **(C)** ΔΨ_m_ foldchange over baseline following the addition of Oligomycin, in physiological and CI impairment conditions (CI_80%_). Individual simulations (dots) are coloured relative to PC simulations (see Methods): White: Within ±10% of PC; Darkening shades of red indicate increase above PC, darkening shades of blue indicate decrease below PC. n = 1000 simulations for each condition.

**Fig 3:**
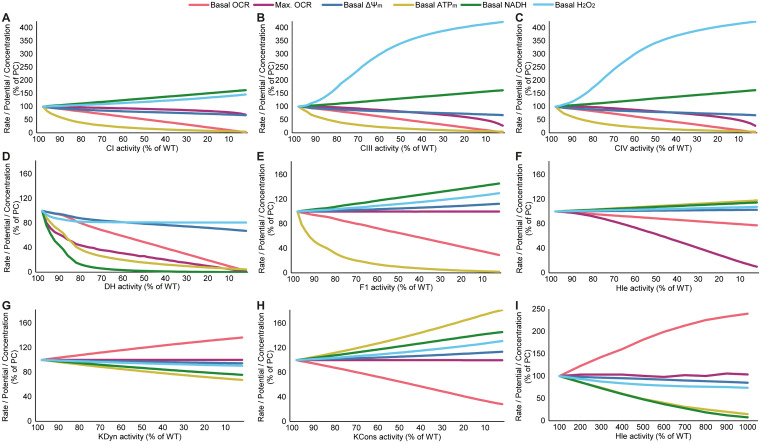
Simulating impairments of critical model components predicts their impact on bioenergetic parameters. The activity of the indicated model components (as a % of the simulated physiological conditions (PC)) is plotted on the x-axis against basal oxygen consumption rate (OCR), maximal OCR, basal mitochondrial membrane potential (ΔΨ_m_, normalised to a min ΔΨ_m_ of −50 mV, for visualisation), and basal concentrations of mitochondrial ATP, NADH, and cytosolic ROS (H_2_O_2_). Impairments were simulated in **(A-C)** ETC complexes I, III and IV [CI, CIII, CIV]; **(D)** the dehydrogenase flux providing substrate to the ETC [DH]; **(E)** the F_1_F_o_ ATP synthase [F1]; **(F, I)** proton leak [Hle]; **(G)** cytosolic ATP production [KDyn]; **(H)** cytosolic ATP consumption [KCons].

**Fig 4:**
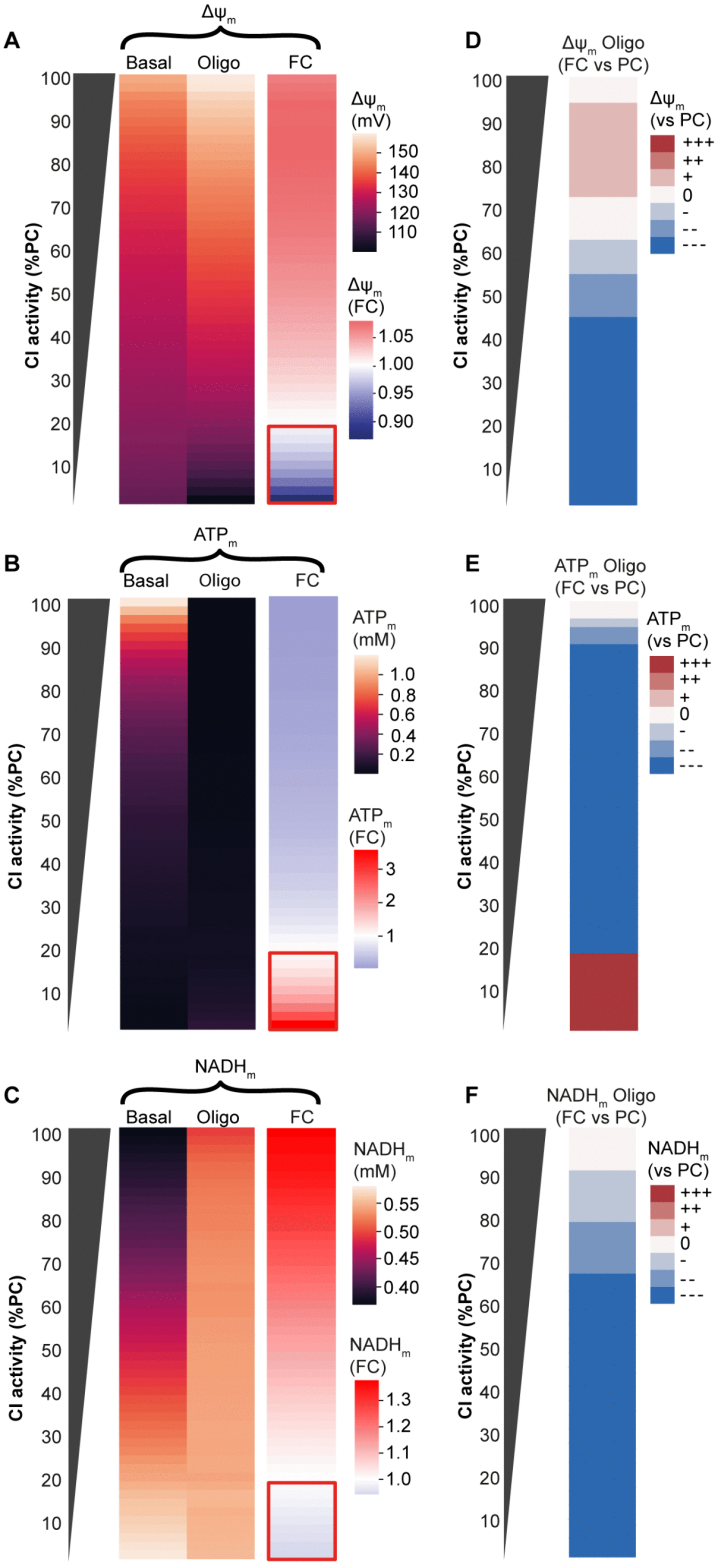
Categorising simulated impairments enables comprehensive analysis of the effects of mitochondrial ETC impairments on key bioenergetic parameters. **(A-C)** Predicted impact of reduced complex I (CI) activity on **(A)** mitochondrial membrane potential (ΔΨ_m_), **(B)** mitochondrial ATP (ATP_m_) levels and **(C)** mitochondrial NADH (NADH_m_) levels at baseline and following the simulated addition of Oligomycin (Oligo; F_1_F_o_ ATP synthase inhibitor). FC: foldchange (compared to basal value). Heatmap values are the median of 50 simulations. The red box indicates F_1_F_o_ ATP synthase reversal predicted in the presence of severe CI defects, as evidenced by a decrease in ΔΨ_m_ following Oligomycin addition. **(B,D,F)** The foldchange (FC) response to Oligomycin is grouped into 7 categories, as described in Methods (decrease---, decrease--, decrease-, no change, increase + , increase++, increase+++ compared to simulated physiological conditions [100% CI activity]). The effect of other impairments is provided in Supp Data.

To model the normal variability that occurs in any cell population, simulations were performed ≥50 times with parameter values and initial concentrations varied within a normally distributed range of +/-20%, as described previously [17]. Individual simulations are visualised in Figs 1–3, and the median of all simulations is shown in Figs 4–. When simulating impaired complex activity (see below), sampled parameter combinations occasionally led to numerical instability (NA output). If this occurred for ≥3 simulations in a population (>5%), the parameter being adjusted was varied by the 4^th^ decimal point (e.g., 0.009 varied to 0.009001) until numerical stability was maintained.

**Fig 5:**
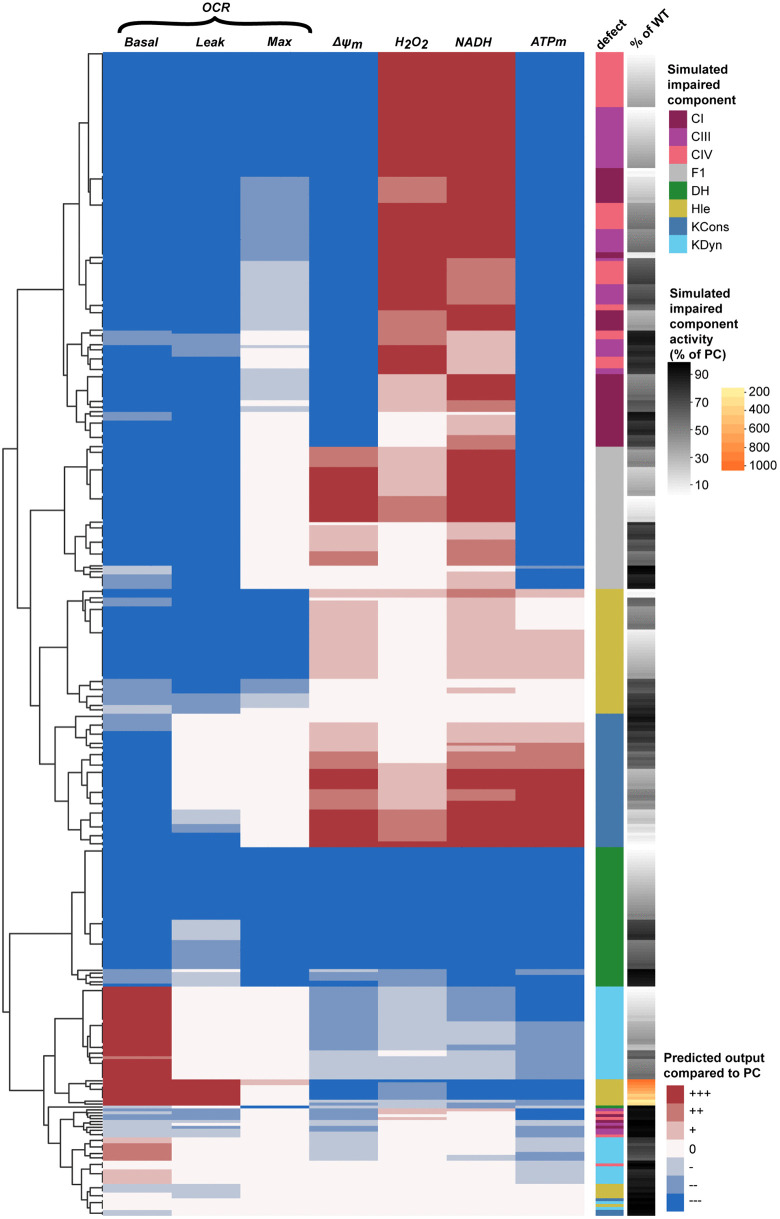
Unsupervised clustering separates impairments according to the induced bioenergetic phenotype. Impairments generally cluster together as they induce distinct bioenergetic phenotypes. Red/blue shading indicates the predicted change in the presence of the simulated impairment compared to physiological conditions (PC) as described in Methods. Row annotations indicate the component with the simulated impairment (CI, complex I; CIII, complex III; CIV, complex IV; F1, F_1_F_o_ ATP synthase; DH: dehydrogenase flux; Hle, proton leak; KCons, cytosolic ATP consumption; KDyn, cytoslic ATP production) and the magnitude of the defect [black-white: 100%−0% of activity in PC]. Leak = oligomycin-insensitive oxygen consumption rate (OCR).

**Fig 6:**
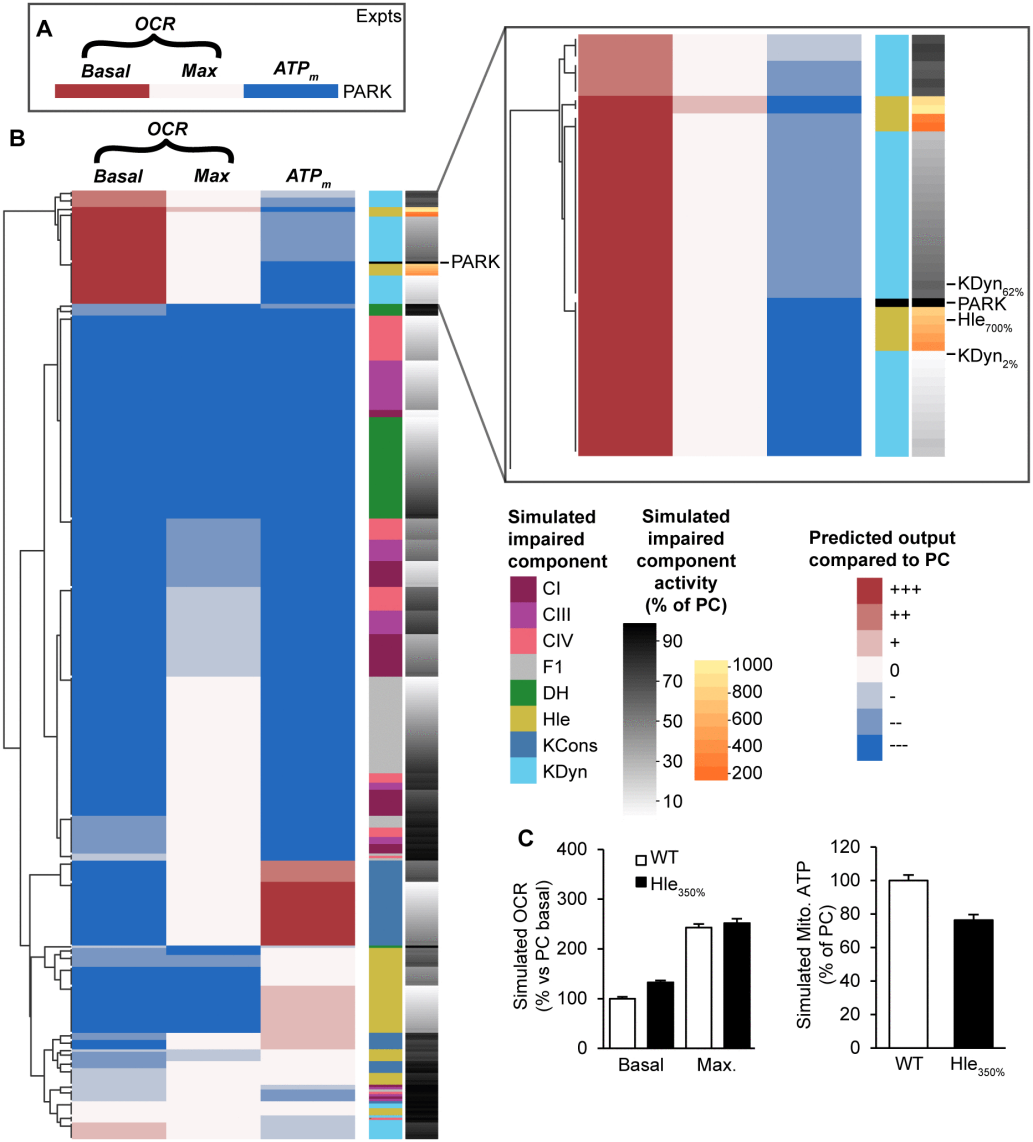
Unsupervised clustering predicts that an increased proton leak may underlie the bioenergetic phenotype measured in Parkin KO dopaminergic neurons (Giguiere et al, 2018). **(A)** Experimental bioenergetic phenotype in Parkin KO dopaminergic neurons (PARK) as measured by [50] – increased basal OCR, unchanged maximal OCR, reduced mitochondrial ATP. (B, inset) The experimental phenotype clustered with a simulated increase in proton leak (Hle), equivalent to increased mitochondrial uncoupling, and large impairments in cytosolic ATP production (KDyn). Red/blue shading indicates predicted change compared to physiological conditions (PC) as described in Methods. Row annotations indicate the component with the simulated defect (CI, complex I; CIII, complex III; CIV, complex IV; F1, F_1_F_o_ ATP synthase; DH: dehydrogenase flux; Hle, proton leak; KCons, cytosolic ATP consumption; KDyn, cytosolic ATP production) and the magnitude of the defect [black-white: 100%−0% of activity in PC]. **(B)** A simulated increase in Hle (350% PC) reproduced the experimental observations [compare with Figs 2A and 2G from [50]].

**Fig 7:**
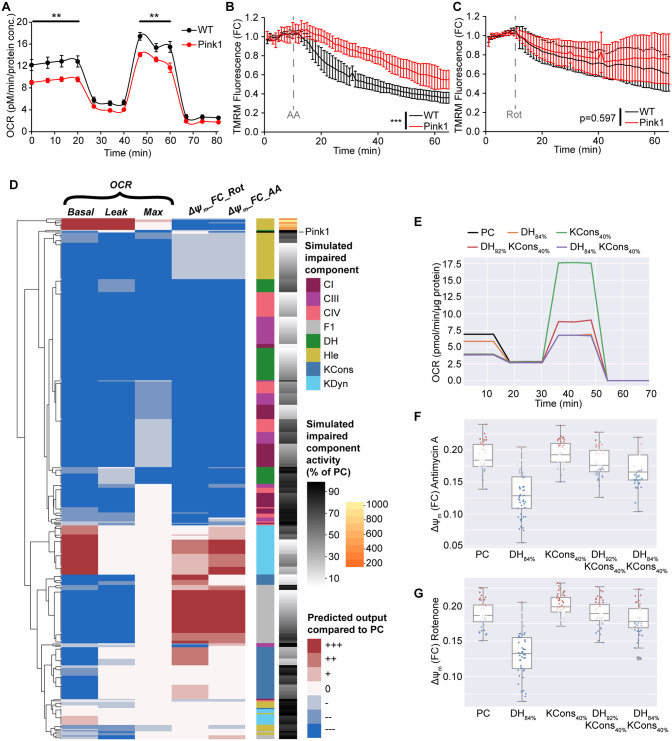
Model-guided semi-automated analysis predicts that combined defects in complex I, F_1_F_o_ ATP synthase and proton leak can mechanistically explain the bioenergetic phenotype observed in Pink1 KO neurons. **(A-C)** Experiments in primary cortical neurons from Pink1 KO mice identified **(A)** significant reductions in basal and maximal oxygen consumption rates (OCR), **p < 0.01, post-hoc comparison; **(B)** reduced ΔΨ_m_ sensitivity to Antimycin A (AA; CIII inhibition), ***p < 0.001, genotype x treatment interaction (pre- vs post-drug), linear mixed effects model; and **(C)** no change in the ΔΨ_m_ response to Rotenone (Rot; CI inhibition; p = 0.597). Data are shown as mean + /-SEM **(A)** and mean + /-st. dev **(B,C)**. **(D)** Unsupervised clustering of simulated and experimental phenotypes demonstrated that while defects in Hle or DH partially reproduced Pink1 KO (Pink1) experimental behaviour, no single defect accurately recapitulated all experiments. **(E-G)** Simulated experiments [**(E)** OCR, **(F)** ΔΨ_m_ foldchange (FC) response to Rotenone, **(G)** ΔΨ_m_ foldchange (FC) response to Antimycin A] demonstrated that the combined impairments of DH_84%_ and KCons_40%_ accurately reproduces the entire set of experiments in Pink1 KO neurons. PC; simulated physiological conditions.

### Simulating drug additions and specific experiments

Oligomycin addition (F_1_F_o_ ATP synthase inhibitor) was simulated by reduction of the x_F1_ parameter to reduce flux through the F_1_F_o_ ATP synthase (F1). FCCP (protonophore) was simulated by increasing the x_Hle_ parameter, modelling an increased proton leak across the inner mitochondrial membrane. Rotenone and Antimycin A (complex I and III inhibitors, respectively) were simulated by reducing the x_CI_ and x_CIII_ parameters. We simulated inhibitor concentrations commonly used in primary neuron experiments (Oligo: 2 μg/ml, Rot: 2 μM, AntiA: 1 μM). For instance, the standard ‘mitochondrial stress test’ performed in respirometry experiments to measure oxygen consumption rate (OCR) [19] can be replicated by simulating sequential addition of Oligomycin, FCCP and Rotenone/Antimycin A. Here, oxygen consumption was assumed as the simulated flux through the oxygen-consuming CIV and the simulated OCR (mol O_2_/s/litre of mitochondria) was converted into experimental units (mol O_2_/min/μg protein) using values for mitochondrial volume (4x10^-14^ l), protein/well (45 μg), and neurons/well (300, 000) as calculated previously [17]. The OCR metrics reported here are ‘basal’ (OCR in baseline conditions), oligomycin-insensitive or ‘leak’ (OCR following the simulated addition of oligomycin) and ‘maximal’ (OCR following the simulated addition of FCCP [Hle increased ~10-fold]). Other experiments, including ΔΨ_m_, NADH, H_2_O_2_ and mitochondrial ATP changes in the presence of the aforementioned inhibitors, were simulated similarly. To compare ΔΨ_m_ model simulations with experiments (Fig 1D), TMRM measurements were converted to mV as previously described [17].

### Local sensitivity analysis: Simulating impaired activity of critical model components

Sensitivity analysis measures the response of output variables to simulated changes in one or several model parameters [32]. Sensitivity analyses on a version of this model was previously performed to rank key parameters affecting bioenergetic state variables in different conditions (*e.g.,* healthy, apoptotic) and to map similarly behaving parameter clusters [33]. To analyse the effect of specific pathology-oriented impairments on mitochondrial bioenergetics, we performed local sensitivity analysis (a single parameter is varied within a defined parameter space) and varied the activity of 8 critical model components – complex I (CI), complex III (CIII), complex IV (CIV), F_1_F_o_ ATP synthase (F1), proton leak (Hle), dehydrogenase flux (DH), cytosolic ATP production (KDyn) and cytosolic ATP consumption (KCons) – by varying their respective model parameters. Activity was varied between 2–100% of the simulated physiological condition (as calibrated to primary cortical neurons in [17]), at 2% intervals. Since component activity varies non-linearly with parameter value, we performed an exponential search to identify parameter values that yield the desired activity (Table 1).

**Table 1:** Sensitivity analysis parameter settings: Model components varied throughout this study, and the corresponding parameter values to simulate the range of impaired/increased activity. PC: simulated Physiological Condition.

Model Component (abbreviation)	Range of simulated activity (% of PC)	Model parameter	Parameter range (unitless)
Complex I (CI)	2-100%	x_CI_	0.80 - 5.75 x10^-5^
Complex III (CIII)	2-100%	x_CIII_	0.36 - 6.78 x10^-4^
Complex IV (CIV)	2-100%	x_CIV_	0.04 - 2.44 x10^-5^
F_1_F_o_ ATP synthase (F1)	2-100%	x_F1_	5.50 x10^-4^–3.86 x10^-7^
Proton leak (Hle)	2-100% 100-1000%	x_Hle_	0.98 - 1.95 x10^-2^ 1 - 10
Dehydrogenase flux (DH)	2-100%	x_DH_	0.75 - 7.55x10^-3^
Cytosolic ATP Production (KDyn)	2-100%	K_ADTP_dyn	0.96 - 9.63x10^-3^
Cytosolic ATP Consumption (KCons)	2-100%	K_ADTP_cons	0.98 - 1.78 x10^-2^

### Categorising quantitative simulated outputs to provide a qualitative representation of model predictions

Power calculations on experimental data from healthy, non-transgenic primary cortical neurons were previously performed to define statistical thresholds beyond which predicted changes in bioenergetic variables are expected to be experimentally measurable [17]. Here, to enable similar thresholding of all model outputs, we first simulated all experiments in physiological conditions (PC) with no defect (n = 1000) to generate the PC distribution (e.g., Fig 2 PC box-plots). We divided this distribution into 7 groups using thresholds at ±10%, ± 25% (Q1, Q3) and ±40% from the median PC value. Subsequently simulated outputs were categorised compared to these thresholds. Outputs within ±10% PC were categorised as ‘no change’. Otherwise outputs were categorised as: decrease-, decrease--, decrease--- (10–25%, 25–40%, > 40% lower than PC, respectively) or increase + , increase++, increase+++ (10–25%, 25–40% > 40% higher than PC, respectively) (Table 2). We estimated that outputs differing from PC by ≥25% (decrease--, decrease---, increase++, increase+++) should be detectable experimentally and that changes that do not exceed these thresholds may not be detectable experimentally. For ΔΨ_m_, NADH, H_2_O_2_ and mitochondrial ATP changes following drug additions, we also calculated the foldchange of the output over baseline, as this is the commonly-reported experimental measurement (these parameters are commonly measured using fluorescent probes, and raw fluorescence measurements should not generally be compared between experiments, due to technical variability) [34].

**Table 2:** Categorising modelled outputs using quantile thresholds from the simulated physiological condition (PC) distribution. Thresholds were defined as ±10%, ± 25% and ±40% change from PC median, corresponding to the 40th/60th, 25th/75th, 10th/90th percentiles of the baseline PC distribution (n = 1000).

Category	Median simulated value compared to simulated physiological condition (PC)	Intepretation
Increase+++	40% above PC	Predicted increase should be detectable in experiments
Increase++	25-40% above PC	
Increase+	10-25% above PC	Model-predicted change may not be detectable experimentally
No change	Within ±10% of PC	Model predicts no meaningful change
Decrease-	10-25% below PC	Model-predicted change may not be detectable experimentally
Decrease--	25-40% below PC	Predicted decrease should be detectable in experiments
Decrease---	40% below PC	

### Hierarchical clustering

To identify defects that induce similar bioenergetic phenotypes, hierarchical clustering was performed in Python with the scikitlearn/seaborn libraries [35,36]. For clustering of simulated data, quantitative modelling outputs were transformed into the qualitative categories described above (increase+++ etc.), categories were assigned numerical values (+3, + 2, + 1, 0, −1, −2, −3) compared to simulations in physiological conditions, and clustering was performed. Cluster quality was assessed by silhouette score and the separation between defects was assessed by V-measure score (as clustering was performed on simulated defects of pre-defined model components, the ground truth labels were known). The choice of linkage method did not affect cluster quality metrics in the case of qualitative inputs. For clustering of both experimental and simulated data, experimental data were assigned values (+3, + 2, + 1, 0, −1, −2, −3) and clustering was performed identically. If the experimental phenotype clustered with the predicted phenotype of a simulated defect, this suggested that this defect may underlie the experimental model.

### Experimental methods

#### Primary cortical neurons.

Experiments involving animals conformed to the guidelines set forth by the Canadian Council on Animal Care (CCAC) and the Canadian Institutes for Health Research (CIHR). All animal procedures were approved by the University of Ottawa Animal Care Committee (breeding and dissection protocol # NSI 1775 and NSI 2459, respectively). The germ line-deleted *Pink1* KO mice were obtained from Dr J. Shen and were backcrossed to C57BL/6 for more than seven generations, as described previously [37]. Primary neuronal cultures were obtained from cortices dissected from backcrossed transgenic *Pink1* (C57BL/6) KO mouse embryos at day E14.5-15.5, as previously described [38]. Wild-type cortical neurons were obtained from embryos of age-matched pregnant dams, which were generated by pairing wild-type offspring derived from heterozygous DJ-1 matings. These timed-pregnant females were euthanized at E14.5–15.5. Pregnant dams were euthanised via intraperitoneal injection of Euthanyl (65 mg/mL) followed by cervical dislocation. Embryos were promptly harvested, decapitated, and cortices dissected. As all procedures were terminal, no additional anaesthesia or analgesia was required. Suffering was minimised through refined handling, adherence to humane endpoints, and limiting time spent outside purpose-built facilities. Experiments were performed after 8–10 days *in vitro* (DIV).

#### Seahorse respirometry.

Live oxygen consumption rates (OCR) were measured using a Seahorse XF24 Analyzer (Agilent). The classical “mitochondrial stress test” protocol was performed as previously described [19], in media including 25 mM glucose and 1 mM pyruvate. OCR was measured in 3–4 wells per condition in 3 independent preps, and normalised to protein concentration in each well, measured using a protein assay kit (Bio-Rad; Cat #500006). Non-mitochondrial OCR was subtracted. The mean OCR for each phase (Basal, Oligomycin-insensitive/ Leak, and Maximal) was calculated and significance was tested using a linear mixed-effects model [lme4 package in R [39]] with genotype, phase, and their interaction as fixed effects, and sample as a random effect. Type II ANOVA was used to assess significance of fixed effects, and post-hoc comparison for each phase was performed using estimated marginal means [emmeans package [40]] with Bonferroni correction for multiple comparisons.

#### TMRM fluorescence.

TMRM fluorescence (10 nM) was measured in live, single neurons following standardized protocols [19]. Rotenone (2 μM) or Antimycin A (1 μM) were added after 10 minutes, and Oligomycin (2 μg/ml) was added after 30 minutes. FCCP (10 μM) was added after 70 minutes to completely depolarize the mitochondrial membrane. Raw fluorescence intensities were normalised to baseline values (average signal intensity in the first 8 minutes prior to drug addition). Fluorescence was measured in n = 20 cells (10 wildtype (WT), 10 *Pink1* KO) from 2 independent preps for Antimycin A experiments, and from n = 37 cells (19 WT, 18 *Pink1* KO) from 3 independent preps for Rotenone experiments. Based on variability in WT cells, these samples sizes provide 90% power (α = 0.05) to detect standardised effect sizes ≥0.135 (Antimycin A) and ≥0.13 (Rotenone). Significance was assessed using a linear mixed-effects model (lme4) with genotype, time and treatment period (pre- vs. post-drug) as fixed effects including all interactions, and random intercepts for individual cells to account for repeated measures. Type II ANOVA was used to assess significance of fixed effects.

## Results

### Computational model of the mitochondrial ETC: Simulating mitochondrial experiments reproduces experimentally-observed behaviours

We aimed to develop a computational pipeline to aid in the interpretation of mitochondrial bioenergetics experiments, and to predict molecular defects underlying dysfunction. We utilised a computational model (Methods, Fig 1A) that describes the mitochondrial electron transport chain (ETC; CI, CIII, CIV), mitochondrial ATP production (F_1_F_o_ ATP synthase and proton leaks), transport across mitochondrial membranes, a coarse-grained model of cytosolic ATP consumption/ production [31] and ETC-mediated H_2_O_2_ metabolism [26]. The model input is provided by a dehydrogenase flux (DH), encompassing NADH/substrate supply upstream of the mitochondrial ETC. Simulated outputs include oxygen consumption rate (OCR), cytosolic/mitochondrial ATP, mitochondrial membrane potential (ΔΨ_m_), mitochondrial NADH and H_2_O_2_ concentration. The addition of pharmacological agents can be simulated using this model, enabling the replication of common experiments (see Methods).

We previously calibrated the model to wildtype primary cortical neurons and to literature, prioritising brain/neuronal data [17,26]. Here, we show that simulating the classical mitochondrial stress test (basal OCR, oligomycin insensitive or ‘leak’ OCR, and maximal OCR) in normal physiological conditions (PC; Fig 1B) closely resembles respirometry measurements in wildtype primary cortical neurons (Fig 1C). The behaviour of other parameters during this experiment can also be visualised (ΔΨ_m,_ mitochondrial ATP, NADH and H_2_O_2_ are shown in S1 Fig). Simulations of Rotenone (Complex I inhibitor), Antimycin A (Complex III inhibitor) and Oligomycin (F_1_F_o_ ATP synthase inhibitor) exposure also correctly predict the changes in mitochondrial membrane potential (ΔΨ_m_) and NAD(P)H, as measured in wildtype primary cortical neurons (Fig 1D-1G). Further comparison of model performance to experimental data is provided in [17,26].

### Simulating impairments of critical model components provides mechanistic insight into experimentally-observed mitochondrial bioenergetic dysfunction

The impact of ETC dysfunction on bioenergetic parameters has been studied experimentally. Impaired activity of ETC complexes (CI, CIII, CIV), for instance, is known to reduce basal OCR [2,41], while increased proton leak elevates basal OCR to maintain the proton gradient [42]. To investigate if the model can reproduce these behaviours, we simulated dysfunction in model components and analysed model outputs. Firstly, we confirmed that a simulated CI impairment indeed decreases basal OCR, while an increase in proton leak causes an elevated basal OCR (Fig 2A). As a second example, we looked at the ΔΨ_m_ response to Oligomycin in physiological conditions (PC) and in the presence of CI impairment (CI_80%_; Fig 2B,C). Under physiological conditions, Oligomycin slightly increases ΔΨ_m_ (Fig 2B left-two box-plots), consistent with its known inhibitory effect on the F_1_F_o_ ATP synthase, and consequent H^+^ gradient retention. In CI-impaired simulations, basal ΔΨ_m_ is strongly reduced compared to PC, while Oligomycin still causes a small increase (Fig 2B right two box-plots). Calculating the foldchange of this response (as commonly done in experiments) demonstrates that the Oligomycin-induced response is predicted to be slightly stronger in conditions of CI impairment (Fig 2C).

To characterise the overall effects of specific ETC dysfunctions, we simulated graded impairments of model components, representative of processes that occur during neurodegeneration. We simulated impaired ETC activity as reported in several neurodegenerative diseases [13,15,16] by reducing the activities of complex I (CI), complex III (CIII), complex IV (CIV), or the F_1_F_o_ ATP synthase (F1), and by increasing/decreasing proton leak (Hle). We modelled impaired substrate supply by reducing the input dehydrogenase flux (DH), and defective cytosolic ATP metabolism by altering cytosolic ATP production (KDyn) or consumption (KCons). We plotted the resulting changes in key bioenergetic parameters, focussing on those that are commonly measured experimentally – basal OCR, max. OCR, basal ΔΨ_m_, basal mitochondrial ATP (ATP_m_), basal NADH and basal H_2_O_2_ (Fig 3). In the presence of these impairments, the model predicted distinct behaviours that agree with current knowledge of cellular bioenergetics. Impairments in ETC complexes (CI, CIII, CIV; Fig 3A-3C) were predicted to reduce basal and maximal OCR and consequently deplete ATP_m_, while NADH levels increased due to reduced NADH consumption [2,41,43]. As ETC complexes pump protons across the mitochondrial inner membrane to maintain the proton gradient, impaired complex activity is predicted to depolarise the mitochondrial membrane potential (ΔΨ_m_). The effect of these impairments on electron transport also increases H_2_O_2_, and the H_2_O_2_ increases predicted upon reduced CIII/CIV activity is > 2-fold higher than that induced in the presence of CI impairments, as reported previously [26,44,45].

In simulations of impaired substrate supply to the ETC (DH; Fig 3D), the model similarly predicted reduced basal OCR, maximal OCR and ATP_m_, and ΔΨ_m_ depolarisation. In contrast to ETC complex inhibition, reduced DH flux substantially depletes NADH levels, as the DH flux encompasses all NADH-generating processes upstream of the ETC. Simulating impaired ATP synthase activity (F1; Fig 3E) accurately predicts ATP_m_ depletion, ΔΨ_m_ hyperpolarisation [as the F_1_F_o_ ATP synthase normally consumes the proton gradient [1,19]], and consequently a reduction in basal OCR with a corresponding NADH increase, due to decreased consumption. Maximal OCR is minimally affected by impaired F1 activity, as the capacity of the ETC is driven by substrate supply and ETC complex activity [1].

Proton leak (Hle) may be either increased or decreased in pathology [3]. Simulated reductions in Hle (Fig 3F) are predicted to decrease basal and maximal OCR – as maximal OCR is induced by addition of uncoupling agents such as FCCP to increase proton leakage into the mitochondria, a reduction in the simulated Hle flux alters this response. Hle reductions slightly increase NADH and ATP_m_ levels, due to increased efficiency of the ETC (more of the proton gradient is available to the F_1_F_o_ ATP synthase for ATP production), and minimally affect ΔΨ_m_. In contrast, increased Hle (Fig 3I) increases basal OCR to maintain the proton gradient (although ΔΨ_m_ is still depolarised), leading to NADH depletion due to increased consumption, and ATP_m_ depletion as the proton gradient is consumed by Hle rather than available to the F_1_F_o_ ATP synthase for ATP production [42].

Finally, impairments in cytosolic ATP production (KDyn; Fig 3G) induce a compensatory increase in basal OCR to maintain cellular ATP, with a corresponding decrease in NADH and slight ΔΨ_m_ depolarisation. ATP_m_ is consequently depleted as more ATP is transported to the cytosol. In contrast, reductions in cytosolic ATP consumption (KCons; Fig 3H) decrease basal OCR due to reduced requirements for ATP production, with a corresponding increase in ATP_m_ and NADH concentrations, and hyperpolarisation of ΔΨ_m_.

The broad effect of severe simulated impairments is summarised in Table 3, and these data further demonstrate the agreement of the computational model with established knowledge of ETC function. Table 3 and Fig 3 can already be used to interpret experimental data – if a decrease in NADH is measured, for instance, this may be caused by an impaired substrate supply to the ETC (DH), an increase in proton leak (Hle), or impaired cytosolic ATP production (KDyn).

**Table 3:** Summary of the predicted changes in bioenergetic parameters (rows) in the presence of severe impairments in the indicated model components (columns): These predictions agree with established bioenergetic knowledge, further highlighting the accuracy of the model. Blue = decrease, white = no change, red = increase. Abbreviations: CI, complex I; CIII, complex III; CIV, complex IV; DH: Dehydrogenase flux, F1: F_1_F_o_ ATP Synthase, Hle: Mitochondrial proton leak, KCons: Cytosolic ATP consumption, Kdyn: Cytosolic ATP production. Down arrow indicates decreased activity of the indicated component, up arrow indicates increased activity.

	↓CI	↓CIII	↓CIV	↓DH	↓F1	↓Hle	↑Hle	↓KCons	↓KDyn
OCR (basal)									
OCR (maximal)									
Δψ_m_ (basal)									
NADH (basal)									
ATP_m_ (basal)									
H_2_O_2_(basal)									

### Categorising simulated responses provides a user-friendly resource to interrogate the effects of mitochondrial ETC dysfunction

To further analyse the effects of these impairments, we can visualise model outputs in heatmap format (Fig 4). As an example, we again looked at the effect of Oligomycin on ΔΨ_m_ in physiological conditions and in the presence of CI impairments (Fig 4A; the effect of other impairments is provided in Supp Data). The heatmap illustrates that Oligomycin is predicted to hyperpolarise ΔΨ_m_ [foldchange (FC) > 1] (as in Fig 2B, 2C), but that in the presence of severe CI defects Oligomycin will depolarise ΔΨ_m_, indicating ATP synthase reversal (red box in Fig 4A). This has been demonstrated experimentally [46,47]. The model also predicts, in agreement with described ETC function, that Oligomycin generally collapses ATP_m_, by inhibiting ATP synthase (Fig 4B). In conditions of ATP synthase reversal however, the ATP synthase consumes ATP and Oligomycin will therefore increase ATP_m_. (red box in Fig 4B). Looking at mitochondrial NADH, Oligomycin increases NADH_m_ due to reduced NADH consumption through CI, and this effect is smaller in the presence of a CI impairment (Fig 4C). Upon ATP synthase reversal, Oligomycin decreases NADH due to increased oxidation.

We next wondered whether changes predicted by the model would be detectable in experiments. We compared simulations in disease conditions and physiological conditions, and categorised responses into seven groups: decrease---, decrease--, decrease-, no change, increase + , increase++, increase+++ (see Methods). We estimated that simulations differing from physiological conditions by ≥25% (decrease--, decrease---, increase++, increase+++) should be detectable experimentally. Applying these categories provides a robust qualitative demarcation and clear visualisation of the impact of defined impairments. It also aids in the interpretation of non-obvious experimental behaviour. Visualising the above-described outputs in this way (Figs 4D-4F) demonstrates that the ΔΨ_m_ response to Oligomycin will be slightly larger when CI is mildly impaired (≥70% activity) but smaller in the presence of severe CI defects (CI < 50%) (Fig 4D). Increases in ATP_m_ upon Oligomycin addition indicate ATP synthase reversal (Fig 4E), while the NADH_m_ response to Oligomycin is graded according to the magnitude of the CI impairment (Fig 4F).

To provide a comprehensive resource to investigate the effects of mitochondrial bioenergetic impairments, we simulated all experimental parameters described in this paper, in the presence of all impairments, and provide all outputs in an Excel file (Supp Data).

### Unsupervised clustering enables semi-automated prediction of molecular defects underlying experimentally-observed bioenergetic phenotypes

We next combined the simulated impairments of all major model components and performed unsupervised clustering. Because impairments that induce similar bioenergetic phenotypes will cluster together, this approach enables us to visualise and identify impairments that induce similar or distinct phenotypes. For this, we analysed OCR parameters from the classical mitochondrial stress test (basal, oligomycin insensitive or ‘leak’, and maximal OCR), as well as baseline levels of ΔΨ_m_, H_2_O_2_, NADH, and mitochondrial ATP (Fig 5). We observed that impairments in F1, DH, Hle, KDyn and KCons tended to cluster separately, indicating distinct bioenergetic phenotypes induced by these impairments. Reduced DH activity, for instance, is characterised by relatively strong decreases in all parameters. In contrast, impairments in CI, CIII and CIV are predicted to induce more similar phenotypes, as flux through the ETC complexes are highly correlated in unimpeded conditions [48], and the effects of these impairments may not be distinguishable in these settings. Nevertheless, mild defects in CI clustered away from defects in CIII and CIV, primarily due to smaller effects on H_2_O_2_ and maximal OCR. Such visualisations can be useful to quickly determine the combined effect of any impairment on the selected bioenergetic parameters.

### Integrated analysis verifies that a bioenergetic phenotype in Parkin knockout dopaminergic neurons can be explained by partial mitochondrial uncoupling

Disease-associated bioenergetic phenotypes are commonly reported in the scientific literature, but identifying the molecular defect underlying such phenotypes may not be straightforward. Our analysis above suggested that integrating experimentally-measured bioenergetic data (‘experimental phenotype’) into our modelling pipeline could predict the molecular defects responsible for the phenotype. By clustering the experimental data with simulated defects, we can identify which impairments produce similar bioenergetic profiles, thereby providing mechanistic insights and inferring potential underlying causes of the observed experimental phenotype. As proof of concept, we utilised experimental data in primary cortical neurons from a transgenic Alzheimer’s mouse model [17], where we previously measured no change in basal or leak OCR, and a reduced maximal OCR (S2A Fig in S2 Fig). We integrated this experimental phenotype with simulated data in the presence of all impairments, and performed unsupervised clustering. The experimental phenotype clustered with a predicted mild defect in DH (S2B Fig in S2 Fig) suggesting that a mild defect in dehydrogenase flux (*i.e.* an impaired substrate supply) would reproduce the experimental phenotype. This agreed with subsequent experiments performed by the authors that identified reduced substrate supply to the ETC [17]. This use-case confirmed our approach, that unsupervised clustering of experimental data with model simulations can pinpoint molecular defects underlying experimentally-measured bioenergetic phenotypes.

We next applied the integrated pipeline to explore the bioenergetic phenotype induced by knockout of Parkin, a PD-related mitophagy gene known to affect mitochondrial Giguiere and colleagues previously measured increased basal OCR and unchanged maximal OCR in dopaminergic neurons from the *substantia nigra* of Parkin knockout mice, but a decrease in ATP content (Fig 6A) [50]. The authors hypothesised that this phenotype may be due to partial mitochondrial uncoupling. We clustered this experimental phenotype with simulated OCR and ATP_m_ outputs for all defects (Fig 6B). In this instance, the experimental phenotype (‘PARK’) clustered with a simulated increase in proton leak (Hle), representing increased mitochondrial uncoupling. The experimental phenotype also clustered with a simulated decrease in cytosolic ATP production (KDyn), providing an alternative molecular explanation, although severe KDyn defects (KDyn < 20% PC) are required to reproduce the experimental phenotype. Mechanistically, these effects are explained in Figs 3I,3G (increased Hle and reduced KDyn, respectively) and associated text. Indeed, subsequent simulation of OCR and ATP_m_ in the presence of increased proton leak (Hle = 350% PC) reproduced the authors’ experimental observations [compare Fig 6C with Figs 2A and 2G from [50]], and verified that the phenotype in Parkin-knockout neurons can be explained by increased mitochondrial uncoupling.

### De novo experiments from Pink1 knockout neurons identify impaired mitochondrial respiration, and the integrated pipeline predicts that multiple defects may underlie this bioenergetic phenotype

PINK1, a serine/threonine kinase localised to mitochondria, is implicated in rare inherited forms of PD [51]. Along with PARKIN, PINK1 is known to play a key role in mitochondrial quality control, and knockout (KO) of the *Pink1* gene affects mitochondrial function, impairs CI activity and increases oxidative damage [49,52,53]. We next utilised primary cortical neurons from *Pink1* KO mice to explore the effect of PINK1 on mitochondrial bioenergetic function. We measured reduced OCR compared to WT conditions (basal, maximal; Fig 7A) and a reduced sensitivity of ΔΨ_m_ to CIII inhibition (Antimycin A; Fig 7B). The response of *Pink1* KO neurons to CI inhibition (Rotenone) was similar to WT neurons (Fig 7C).

To identify the putative molecular defect that may underlie this phenotype, we applied our integrated pipeline as above with the Pink1 KO experimental phenotype (Fig 7D). In this instance, the experimental phenotype did not match the phenotype of any individual simulated defects, but clustered closely to several defects. This predicted that no single defect could explain all the observed data, but that several defects partially explained the experimental observations. Specifically, a reduced proton leak (Hle < 70%) is predicted to decrease all OCR metrics and does not impact the ΔΨm response to Rotenone (Figs 7D). However, in contrast to experiments, it also decreases OCR leak and is not predicted to impact the ΔΨm response to Antimycin A (Figs 7D). A Hle defect is unlikely here, as OCR leak was not significantly decreased in experiments (Fig 7B). Similarly, mild defects in DH decrease OCR metrics and reproduce the decreased ΔΨ_m_ sensitivity to Antimycin A (Figs 7D-7F), but these defects are also predicted to induce a strong decrease in the ΔΨ_m_ FC response to Rotenone (Figs 7D,7G), an effect not observed in experiments.

We therefore hypothesised that a combination of simulated defects may be required to fully explain the experimental data, and utilised model predictions to explore potential combinations. We focussed on mild DH defects (DH > 80%) with minimal impact on OCR leak, but a strong impact on the ΔΨ_m_ FC response to Rotenone (Figs 7E,7G). We noted that impairments in cytosolic ATP consumption (KCons) increased the Rotenone response with minimal impact on other parameters (Figs 7D-7G), and reasoned that combining these two defects (DH, KCons) may reproduce the experimental behaviour. Indeed, simulations identified that these defects together reproduced the experimentally-observed behaviour in *Pink1* KO neurons and may underlie this phenotype (Figs 7E-7G). This would suggest an intact ETC but impairment of linked bioenergetic processes.

## Discussion

We here applied a computational model to investigate mitochondrial bioenergetic dysfunction in the presence of pathophysiological processes that occur during neurodegeneration, and established an analysis pipeline to predict underlying molecular defects that can explain experimentally-observed mitochondrial bioenergetic dysfunction. We also provide an open resource to aid interpretation of complex experimental data, provide mechanistic insight, generate hypotheses, and inform experimental design.

We first simulated several pathological impairments and analysed the predicted effect on key mitochondrial bioenergetic parameters. Simulations agreed with commonly-observed experimental data, including reduced OCR and increased NADH upon ETC complex inhibition, mitochondrial membrane hyperpolarisation following impairment of the F_1_F_o_ ATP synthase [41,43], and reversal of the ATP synthase in the presence of severe ETC complex impairments [1,19]. As the model is a theoretical representation of a complex biological system, the model cannot simulate the expected biological variability as fluxes/concentrations tend to zero. We therefore simulated defects down to 2% of physiological conditions, but not beyond. These simulations demonstrated the accuracy of the model according to established knowledge of mitochondrial ATP bioenergetics, and provides precise mechanistic explanations of experimental behaviour in the presence of several bioenergetic impairments as reported in pathology. These simulations (Fig 3, Table 3) can be used as an aid to interpret mitochondrial bioenergetic experimental data.

Several computational models of ETC function have been developed [*e.g.,* [54–58]]. The computational model used here simulates the ETC complexes as separate entities, rather than the lumped functions in Keizer and Cortassa models [54,55]. However, it maintains reduced complexity compared to other models [*e.g.,* [57,58]] and utilises mass action kinetics, a simplification that cannot capture threshold behaviour or saturation kinetics. We have accounted for this in part by defining analytical thresholds to indicate whether a predicted change would be experimentally detectable. The model does not incorporate, for example, beta oxidation, the effects of alternative substrates, or individual TCA cycle components. Similarly, the phenomenological ROS metabolism equations described here [26] do not explicitly model NAD(P)H-dependent scavenging processes, which may influence redox balance and oxidative stress [59]. While the inclusion of additional details may capture a broader range of biological processes, we have utilised a simplified model to enable faster calibration, lower computational burden, and easier interpretation. By focusing on key relationships and variables, our model provides meaningful and accessible explanations and predictions.

We note that the model cannot represent behaviours outside of the modelled components. A simulated impairment in the dehydrogenase flux input function, for instance, does not distinguish between TCA cycle dysfunction or defects in substrate import to the mitochondria, but rather represents a cumulative impaired supply of substrate to the ETC. We acknowledge that mitochondrial bioenergetic phenotypes occur that cannot be explained by the described model system. Simulation results, as with experimental data [20], should be interpreted carefully. Future work could incorporate more detailed models of ETC complexes, membrane potential and H_2_O_2_ production including Michaelis-Menten kinetics, which will also require calibration to experimental data from neurons.

We demonstrated that clustering simulated outputs can identify impairments that induce similar bioenergetic phenotypes. Thus, by comparing an experimental phenotype with model simulations of known ETC defects, the pipeline predicts specific defect(s) that could explain the experimentally-observed phenotype. This is a novel technique to integrate experimental and computational data, and this approach enabled us to predict molecular defect(s) contributing to the bioenergetic phenotypes observed in *Parkin* and *Pink1* KO neurons. Utilising experimental data from *substantia nigra* dopaminergic neurons in a *Parkin* KO mouse model of PD [50], the pipeline verified that the observed bioenergetic phenotype could be explained by an increase in proton leak (mitochondrial uncoupling), as had been hypothesised by the authors [50], or by a severe decrease in cytosolic ATP production. The model also provides a mechanistic explanation for these effects (Figs 3I,3G) – (i) an increased proton leak induces a compensatory increase in basal OCR to maintain the proton gradient, but this is not sufficient to maintain mitochondrial ATP levels, or (ii) reduced cytosolic ATP production leads to an increase in basal OCR to restore cytosolic ATP, but similarly is not sufficient to maintain mitochondrial ATP. To identify the more probable defect underlying the *Parkin* KO disease state, we reasoned that a severe decrease in cytosolic ATP production is less likely to occur in a biological setting. The pipeline does not predict whether this is a direct or indirect effect of *Parkin* KO, e.g. on mitophagy. A recent report suggests that any impact on ETC function may be indirect [60].

Our pipeline enables interpretation of the bioenergetic phenotype observed in a particular system. As demonstrated, this approach aims to predict the defect that induces the phenotype most similar to the experimental phenotype, rather than computing the exact solution as in a traditional linear or nonlinear least squares data fitting. This means that model predictions can be generalised to other experimental systems with comparable ETC configuration (*i.e.* intact cultured cells respiring predominantly on CI substrates), as the direction rather than the magnitude of change is most important, and is less impacted by noise, system variability or model kinetics/parameterisation. While we focussed here on genetic forms of PD, the pipeline can equally be applied to interrogate sporadic PD or other disorders displaying mitochondrial bioenergetic dysfunction. While the pipeline could also compare cell-type specific bioenergetic phenotypes (e.g. dopaminergic vs cortical neurons) subtle differences in mitochondrial function/dysfunction may not be captured by this qualitative approach. Our pipeline also enables the ranking of putative defects, by reverting to the quantitative simulated data that measures the predicted effect size of each defect, helping to prioritise mechanistic hypotheses for further testing.

Finally, we generated primary cortical neurons from a *Pink1* KO mouse model of PD, and identified reduced OCR capacity and increased resistance to CIII inhibition. We applied our computational pipeline to these *de novo* data and predicted that multiple impairments may be required to completely recapitulate the experimentally-observed bioenergetic phenotype. Simulations helped us to identify that combined impairments in DH (dehydrogenase flux, i.e. an impaired substrate supply) and KCons (cytosolic ATP consumption) may explain the observed experimental phenotype. As we did not simulate all possible defect combinations, we cannot exclude that alternative combinations could also reproduce the phenotype. PINK1 deficiency has been shown to impact substrate availability [61] potentially through TCA cycle alterations [62], and alterations in mitophagy may also have downstream effects on bioenergetics.

In conclusion, we here developed and applied a computational pipeline to interrogate mitochondrial bioenergetics in the presence of PD familial mutations. We verified that increased mitochondrial uncoupling may underlie the bioenergetic phenotype in *Parkin* KO dopaminergic neurons, and that a combination of defects is required to explain the bioenergetic impairments measured in primary cortical neurons from *Pink1* KO mice. We also provide a comprehensive resource (Supp Data) detailing the effects of mitochondrial ETC dysfunction on key bioenergetic parameters. This resource can aid the interpretation of complex mitochondrial bioenergetic experiments, enable hypothesis generation, and inform experimental design.

### Highlights

- The complexity of mitochondrial bioenergetics can make experimental data difficult to interpret.
- We simulated a computational model of mitochondrial bioenergetics in healthy and pathological conditions, and established an analysis pipeline to integrate model simulations with experimental data.
- We applied the pipeline to data from Parkinson’s Disease models to predict the molecular defects underlying Parkinson’s-related pathology.
- We provide all outputs in a user-friendly Excel file, which serves as a valuable resource to the community for insight into the effects of pathology on mitochondrial bioenergetics and for interpretation of experimental results.

## Supporting information

S1 Fig Model simulations during the mitochondrial stress test. Simulations of (A) mitochondrial membrane potential (ΔΨ_m_), (B) mitochondrial ATP, (C) mitochondrial NAD(P)H and (D) H_2_O_2_ during the mitochondrial stress test – simulated addition of Oligomycin, FCCP and Antimycin A. After Antimycin A, mitochondria are depolarised (no flux through ETC) and both ATP_m_ and NADH_m_ are maintained by influx from the cytosol. Traces represent individual simulations (n = 50), with the mean of all simulations shown in black.
(TIF)

S2 Fig Clustering simulated OCR metrics with experimental phenotype from a transgenic Alzheimer’s model (TgAD) confirms reduced substrate supply as the underlying molecular defect. (A) The TgAD experimental phenotype as measured by [17] – unchanged basal and leak OCR, decrease in max OCR. (B, inset) The TgAD experimental phenotype clusters with a simulated mild defect in dehydrogenase flux (DH, 96−98% of wildtype flux). Red/blue shading indicates predicted change compared to physiological conditions (PC) as described in Methods. Row annotations indicate the component with the simulated defect (CI, complex I; CIII, complex III; CIV, complex IV; F1, F1F0 ATP synthase; DH: dehydrogenase flux; Hle, proton leak; KCons, cytosolic ATP consumption; KDyn, cytosolic ATP production) and the magnitude of the defect [black-white: 100%−0% of wildtype (WT) activity]. Leak = oligo-insensitive OCR.
(TIF)
